# Human tissue cultures of lung cancer predict patient susceptibility to immune-checkpoint inhibition

**DOI:** 10.1038/s41420-021-00651-5

**Published:** 2021-09-25

**Authors:** David Junk, Sebastian Krämer, Johannes Broschewitz, Hennig Laura, Chiara Massa, Yousef Moulla, Ngoc Anh Hoang, Astrid Monecke, Uwe Eichfeld, Ingo Bechmann, Florian Lordick, Barbara Seliger, Sonja Kallendrusch

**Affiliations:** 1 Institute of Anatomy, University of Leipzig, Liebigstr. 13, 04103 Leipzig, Germany; 2 Department of Visceral, Transplantation, Thoracic and Vascular Surgery, University Hospital Leipzig, Liebigstraße 20, 04103 Leipzig, Germany; 3 Department of Visceral and Thoracic Surgery, University Hospital Brandenburg, Gehrbelliner Straße 38, 16816 Neuruppin, Germany; 4 Institute of Medical Immunology, Martin Luther University Halle-Wittenberg, 06112 Halle, Germany; 5 University Cancer Center Leipzig, University Hospital Leipzig, Liebigstraße 20, 04103 Leipzig, Germany; 6 Institute of Pathology, University Hospital Leipzig, Liebigstraße 26, 04103 Leipzig, Germany

**Keywords:** Immunosurveillance, Cancer microenvironment

## Abstract

Despite novel immunotherapies being approved and established for the treatment of non-small cell lung cancer (NSCLC), ex vivo models predicting individual patients’ responses to immunotherapies are missing. Especially immune modulating therapies with moderate response rates urge for biomarkers and/or assays to determine individual prediction of treatment response and investigate resistance mechanisms. Here, we describe a standardized ex vivo tissue culture model to investigate individual tumor responses. NSCLC tissue cultures preserve morphological characteristics of the baseline tumor specimen for up to 12 days ex vivo and also maintain T-cell function for up to 10 days ex vivo. A semi-automated analysis of proliferating and apoptotic tumor cells was used to evaluate tissue responses to the PD-1 inhibitor nivolumab (*n* = 12), from which two cases could be successfully correlated to the clinical outcome. T-cell responses upon nivolumab treatment were investigated by flow cytometry and multispectral imaging. Alterations in the frequency of the Treg population and reorganization of tumor tissues could be correlated to nivolumab responsiveness ex vivo. Thus, our findings not only demonstrate the functionality of T cells in NSCLC slice cultures up to 10 days ex vivo, but also suggests this model for stratifying patients for treatment selection and to investigate in depth the tumor-associated T-cell regulation.

## Introduction

NSCLC is responsible for the most cancer-related deaths worldwide and has the highest incidence rate among all tumors [1]. Two main histological subtypes classify the NSCLC, the squamous NSCLC (sNSCLC) and the NSCL adenocarcinoma (NSCLaC). In addition, several molecular subtypes of this disease are identified. Extensive genomic alterations, high mutation loads, and late diagnosis are some major factors responsible for the poor prognosis of patients, with a 5 years’ survival rate of ~17% [2]. Surgical resection alone or combined concepts with chemotherapy and radiotherapy are still the standard therapy for stage I–III NSCLC, partially combined with pretested targeted therapy or immune-checkpoint inhibitors. The most used chemotherapeutic drugs are platin derivatives combined with vinorelbine, gemcitabine or paclitaxel, respectively. In the presence of genetic mutations (EGFR, ALK), patients benefit from treatment with selective EGFR inhibitors (e.g., gefitinib and erlotinib) or the ALK inhibitor crizotinib compared to standard chemotherapy for NSCLC tumors [3, 4]. However, only very few patients are eligible for targeted therapy, examining the mutational burden or other specific predictive markers [5].

The presence of immune cells within tumors already shown by Rudolf Virchow a century ago has become evident and various novel approaches are currently investigated [6]. Targeting the immune system is a highly promising approach for the treatment of cancer, but can lead to life-threatening adverse effects. Recently, a prolonged overall survival (OS) of NSCLC patients was reported upon checkpoint inhibition in the phase I trial checkmate227 [7]. About 20% of the patients with PD-L1 expression levels ≥1% have a 2 months increased cancer survival upon treatment with immune-checkpoint antibodies. However, the underlying mechanisms leading to response or failure of treatment have not yet been identified. A checkmate026 subgroup analysis suggested that the individual mutational burden in NSCLC might predict therapy response, despite predictive tests or strong biomarkers are still missing [7, 8]. Therefore, stable and reproducible methods that allow the prediction of responses are urgently needed to prevent patients from unnecessary treatment, in addition to technologies that offer relevant test platforms for tumor marker investigation and drug testing.

Three dimensional culture systems, like organoids or tissue cultures, offer new opportunities in personalized therapy testing. Tissue cultures have a long tradition and have recently been adapted also to tumor tissue, allowing to study the complexity of the tumor and its microenvironment (TME) as well as its susceptibility to therapeutic interventions [9–17]. Human tissue cultures recapitulate most aspects of the derived tissue, like differentiation, the microarchitecture and the cellular variety [12, 15, 18]. Advantages are the short establishment time and the simple manipulation. In the present work, we established a culture protocol for NSCLC specimen, which could be employed to assess responses to chemotherapy as well as to immune-checkpoint inhibitors.

## Results

### Heterogeneity and morphologic preservation of lung cancer slice

**Fig. 1:**
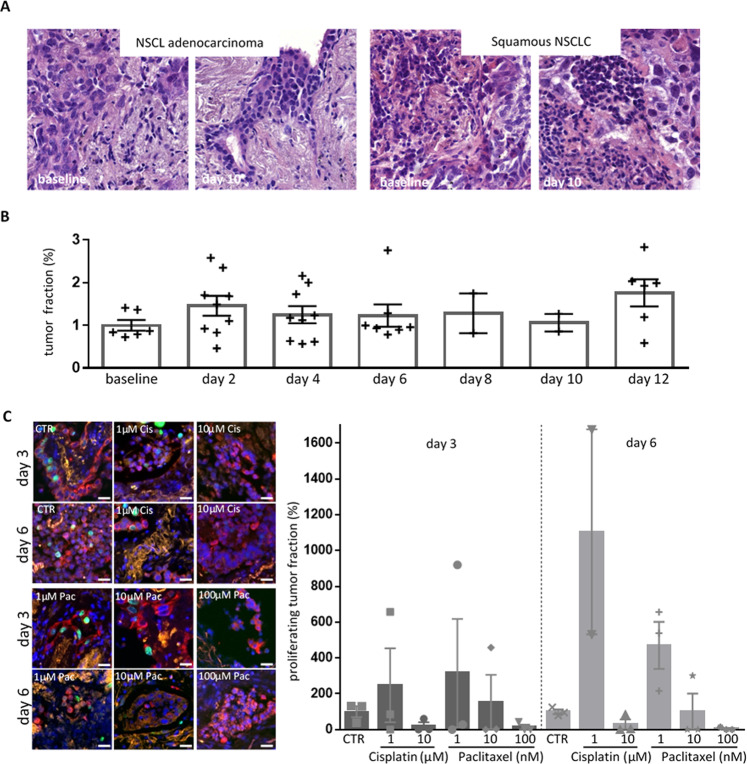
Tissue maintenance and dose-dependent response ex vivo. **A** Slices from tumor specimen were stained with HE at baseline or after culture in control conditions for up to 12 days. Shown are representative HE staining of the two different NSCLC subtypes at baseline and after 10 days ex vivo. **B** The tumor fraction present in the cultured specimens was evaluated and normalized to the baseline. Shown are the results from three different patients. To control reproducibility multiple slides from the same patients were measured at the same timepoint. **C** Slices were cultured for 3 or 6 days with different doses of cisplatin (1, 10 µM) or paclitaxel (1, 10, 100 nM). To calculate the proliferating tumor fractions tissues were stained for Ki67 (green), pancytokeratine (red), and counterstained with Hoechst (blue). Shown are pictures of the different treatment at day 3 and 6 (right) as well as the overall variations in proliferating tumor fraction within the one tissue specimen for which enough material was available to perform all conditions with at least two replicates (left). Background, yellow; Bar = 50 µm.

A tissue culture protocol for cultivation of patient-derived lung cancer specimens for up to 12 days ex vivo was successfully established (Fig. 1). To adjust for differences in auto-fluorescence characteristics and staining intensities, we combined stain-specific sequences of processing and segmentation algorithms in Image J [19]. Slice cultures showed a good preservation of morphologic features of the original tumor (Fig. 1A). Parameters including cell formation, mucin production, and staining characteristics were assessed for comparison from 24 tumor specimens. In all samples, the pathological diagnosis of the original tumor matched the features found in baseline culture, representatively shown in Table 1. 4/24 cases (16,6%) were excluded from the analysis due to high necrotic areas and inflammatory regions observed in the baseline tissue. Specimens not shown in the table were used for adjustments of the culture conditions. As shown in Fig. 1B TFs were maintained up to day 12 ex vivo. The overall TF of 31% ± 12 (*n* = 3, mean ± SD) was nonsignificantly enhanced from day 2 to day 10 up to 53% ± 12 and to 68% ± 14 at day 12.

**Table 1:** Patient specimens.

No.	Age, sex	Type of NSC lung carcinoma	Ex vivo treatment	PD-1	Response ex vivo	Co-culture
1	62, F	Moderate differentiated adenocarcinoma (G2)	Culture over time			
2	58, M	Poor differentiated adenocarcinoma (G3)	Culture over time			
3	61, M	Moderate differentiated adenocarcinoma (G2)	Time/dose-response			
4	73, F	Moderate differentiated adenocarcinoma (G2)	NIVO 5 and 10 day	Negative	NR	x
5	54, M	Poor differentiated adenocarcinoma (G3)	NIVO 5 and 10 day	50%	R	x
6	79, F	Moderate differentiated adenocarcinoma (G2)	NIVO 5 and 10 day	5%	R	x
7	79, M	Moderate differentiated squamous epithelial carcinoma (G2)	NIVO 5 and 10 day	50%	NR	x
8	56, M	Moderate differentiated adenocarcinoma (G2)	NIVO 5 and 10 day	60%	NR	
9	58, M	Moderate differentiated squamous epithelial carcinoma (G2)	T-cell regulation	5%	NR	
10	57, F	Poor differentiated adenocarcinoma (G3)	T-cell regulation	25%	NR	
11	81, M	Moderate differentiated squamous epithelial carcinoma (G2)	T-cell regulation	n.d.	R	
12	57, M	Moderate differentiated squamous epithelial carcinoma (G2)	T-cell regulation	Negative	NR	
13	79, F	Minimal differentiated adenocarcinoma (G1)	T-cell regulation	15%	NR	
14	78, M	Minimal invasive adenocarcinoma (GX)	T-cell regulation	n.d	NR	
15	66, M	Poor differentiated adenocarcinoma (G3)	T-cell regulation	5%	R	

### Effects of cytotoxic drugs on tumor cell proliferation

Slice cultures were treated with different doses of the cytotoxic drugs cisplatin (1, 10 µM) or paclitaxel (1, 10, and 100 nM). The effect on tumor cell proliferation was determined by antibody staining of Ki67. Whereas nonsignificant changes due to high intratumor variances were detected after 3 days of treatment, a significant trend was found after 6 days in culture. Low doses of both drugs resulted in an increased tumor proliferation rate compared to the control condition. In contrast, higher concentrations of both drugs showed a dose-dependent decrease in tumor proliferation (Fig. 1C).

### Response to nivolumab is independent of lymphocyte supplementation in tumor tissue cultures

**Fig. 2:**
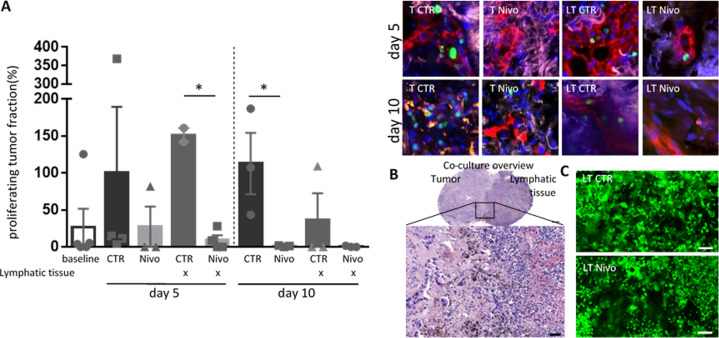
Nivolumab tissue response is independent of lymphocyte supplementation. NSCLC tissue cultures were cultivated alone (T) or with autologous lymphatic tissue (LT) in the presence or absence of Nivolumab for 5 or 10 days. **A** Percentages of Ki67 proliferating tumor fractions in the different culture conditions from one patient specimen are shown upon normalization to the control culture in the absence of lymphocytes (left) Kruskal–Wallis test showed significant differences. Representative pictures of the different conditions at day 5 and 10 are shown (right). pancytokeratine, red; Ki67, green; Hoechst, blue; background, white. Bar = 50 µm. **B** Shown is the HE staining of a NSCLC tissue co-cultured with lymphatic tissue slice at day 10. Overview, bar = 500 µm; HE magnification, bar = 50 µm. **C** Calcein live staining (green) of a control tissue (top) and a nivolumab treated tissue (bottom) after 5 days in co-culture with lymphatic tissue. Bar = 200 µm.

To investigate nivolumab susceptibility, the presence of T-lymphocytes in the cultivated tissue is required. Therefore, we evaluated whether immune cells still present in the NSCLC tissue would be sufficient to detect an effect of the immunotherapy or if an “exogenous” provision using lymphatic tissue would be required. The co-culture of NSCLC with autologous lymphatic tissue in the absence of additional treatment did not result in a significant reduction of the tumor cell proliferation at day 5 and day 10 (Fig. 2A). In contrast, nivolumab supplementation caused a decreased proliferation of TF in the presence as well as in the absence of lymphatic tissue. This effect was already detected at day 5, but was more pronounced at day 10 ex vivo (Fig. 2A). The HE staining of the co-culture in Fig. 2B demonstrated a high similarity with tissue of the tumor area. A reduced proliferation in the presence of nivolumab with respect to the control condition (Fig. 2A) was accompanied by a diminished number of tumor cells displaying a reduced metabolism, as shown by the calcein live staining (Fig. 2C).

### Heterogeneous response of tumor tissues to therapy

**Fig. 3:**
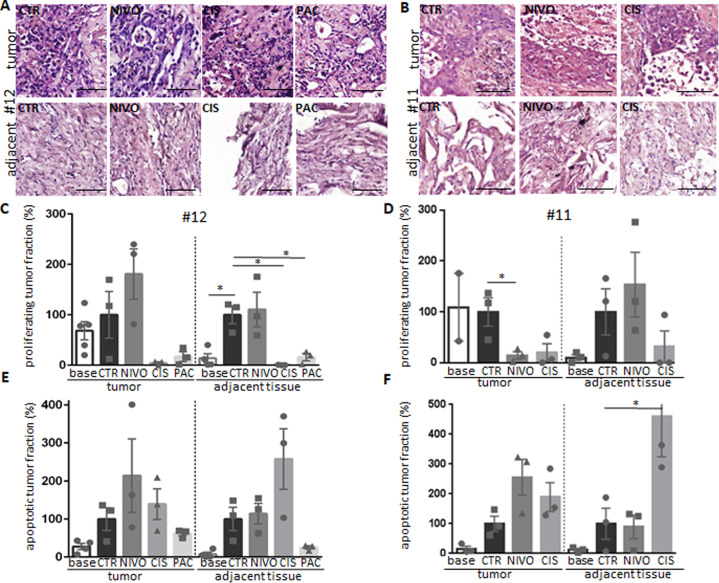
Individual susceptibility in squamous cell carcinoma of the lung and in adjacent tissue. Tissue specimens were treated for 5 days with nivolumab (3 µg/ml), cisplatin (10 µM), or paclitaxel (100 nM, only #12), depending on tissue availability. Shown are representative outcome from patient #12 that did not respond to nivolumab ex vivo (left) and patient #11 that responded (right). Representative HE sections of the tumor as well as of the adjacent tissue of #12 (**A**) and #11 (**B**) at 6 days in culture are shown. Bar = 100 µm. Tumor tissue and adjacent healthy epithelial tissue were evaluated for proliferation (**C, D**) as well as for apoptosis (**E, F**). The adjacent tissue of #12 was not free of tumor cells but only tumor-free areas were evaluated. *p < 0.005 in one-way Anova tested against the control condition. Base baseline.

The individual susceptibility of different sNSCLC samples upon application of cisplatin, nivolumab, or paclitaxel was investigated by determination of proliferation and apoptosis (Fig. 3). Adjacent tissue served as a control in order to determine potential harming effects of the different treatments on the healthy epithelial tissue. HE sections are shown in Fig. 3A and B. In the tumor fraction, treatment with cisplatin or paclitaxel reduced the percentage of Ki67+ cells (Fig. 3C and D, left portion) and induced apoptosis. (Fig. 3E and F, left portion). Interestingly, the culture control conditions induced the proliferation as well as an enhanced apoptosis in healthy epithelial cells of the adjacent tissue (Fig. 3C–F). Both chemotherapy treatment reduced the “culture-induced” proliferation whereas the apoptotic rate was further augmented by cisplatin application in contrast to paclitaxel that induced much lower apoptosis than control conditions (Fig. 3C–F, right portion). Evaluation of the effect of immunotherapy with nivolumab highlighted responder and nonresponder tissue culture: In patient #12 tumor cells still proliferated despite an increased apoptosis induction (Fig. 3C and E), whereas in patient #11 apoptosis induction was also paralleled by a significantly reduced frequency of proliferating tumor cells (Fig. 3D and F). The effects of nivolumab on the adjacent tissue did not significantly differ to that from control conditions (Fig. 3C–F, right portion)

### Unspecific effects of nivolumab by applying an IgG4 antibody

**Fig. 4:**
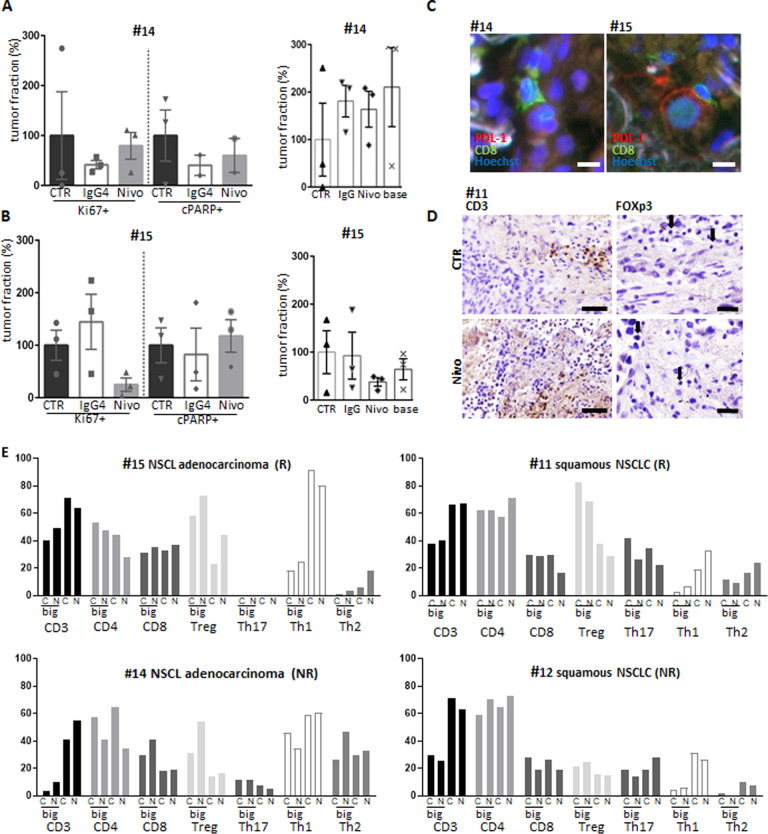
Altered T-cell repertoire after nivolumab treatment in NSCLC. **A, B** Tissue specimens #14 (**A**) and #15 (**B**) were cultured ex vivo under control condition or upon addition of nivolumab or its isotype control, an IgG4 Ab. After 5 days of treatment the tissue were stained for Ki67 or cleaved PARP to evaluate the percentage of proliferating or apoptotic cells as well as for the total fraction of tumor within the tissue. Kruskal–Wallis test was performed. **C** Representative staining of tissue from #14 and #15 treated with nivolumab and stained with PD-L1 (red) and CD8 (green), Hoechst, blue; background, white. Bar = 20 µm. **D** Representative staining of #11 with CD3 (left) or FoxP3 (right) in control (top) and nivolumab (bottom) treated conditions. CD3, bar = 50 µm, FoxP3, bar = 20 µm. **E** Single-cell suspension obtained from the tissue specimens, cultured for 5 days in the presence or absence of nivolumab, were evaluated by flow cytometry. Shown are the percentages of the different T-cell populations as obtained with the gating strategy shown in supplementary Fig. 1. NR nonresponder, R responder.

To have deeper insights into the specific effect of nivolumab, exemplary samples were treated with nivolumab or with the IgG4 isotype control Ab. As shown in Fig. 4A, the tissue of patient #14 was neither significantly affected by nivolumab nor by the isotype control treatment regarding proliferation, apoptosis, the percentage of proliferating cells, or the total tumor fraction. In contrast, nivolumab, but not the IgG4 Ab reduced total tumor fraction as well as the percentage of proliferating tumor cells in specimen #15 (Fig. 4B).

### Altered composition of the tumor microenvironment in response to nivolumab

The responses of the T-cell populations to nivolumab treatment ex vivo was investigated using conventional immunohistochemistry (IHC), multicolor flow cytometry and multispectral imaging. Immunohistological stainings for CD3, CD4, CD8, and FoxP3 was done for consecutive tissue cuts in #11, #12, #14, and #15. In all stainings, lymphocytes differed in size and their distribution within the stromal or tumoral region. Double staining for CD8 and PD-L1 indicated that the nonresponding tissue was PD-L1 negative and the responding tissue PD-L1 positive (Fig. 4C). Staining for CD3 together with Foxp3 indicate that CD3+ cells maintained the distribution pattern and morphological characteristics of the native tissue and showed an invasion into the tumor site after nivolumab treatment. Foxp3+ T cells were observed in tumor stroma as well as in tumor regions. However, different sizes of lymphocytes were observed (Fig. 4C and D).

In order to obtain quantitative information, the tissues were also disrupted and the obtained single-cell suspension was evaluated by flow cytometry. Out of seven experiments, we obtained two tissues responding towards nivolumab treatment, one squamous cell carcinoma and one adenocarcinoma. Five nonresponding specimens (three adenocarcinomas and two squamous cell carcinomas) were also evaluated by flow cytometry, with three of them that did not show any alterations upon treatment and are consequently not shown. In parallel to the histological differences detected in the size of the immune infiltrate, one could differentiate between a small lymphocyte fraction and a fraction that displayed enhanced granularity and size within the forward versus sideward scatter plot. The gating strategy is provided in supplementary Fig. 1.

The cellular fractions of the flow cytometric analysis are shown in Fig. 4E. The NSCLaC tissue (#15) responding to nivolumab treatment showed a decreased CD3, CD4, and Th1 population, while the Treg and Th2 populations were increased within the small lymphocytes. On the contrary, there was an increase in the CD3 and Th1 population within the big lymphocyte population as well as in the Treg cells. The nonresponding NSCLaC tissue (#14) revealed an increase in the CD3 population and a decrease in the CD4 population within the small lymphocytes. In the big lymphocyte gate, nivolumab treatment increased the population of CD3+ cells up to 10% with a prevalent expansion of CD8, Treg, and Th2 cells. The nonresponding sNSCLC tissue (#12) displayed significant lower amounts of CD3 positive cells within the big lymphocyte population (CTR: 30%; nivolumab: 26%) than in the small lymphocyte fraction (CTR: 71%; nivolumab 64%). In both lymphocyte fractions the CD4 population increased, while the CD8 population decreased. In addition, the small lymphocyte fraction showed an increase of the Th17 population, while the Th1 population decreased. An increase of the CD4, Th1, and Th2 population as well as lower levels of CD8, Treg, and Th17 cells were observed in the responding #11. The big lymphocyte population showed only decreased numbers of Treg and Th17 cells. The IgG4 conditions in #14 and #15 demonstrated only minor adaptations compared to the control condition (data not shown).

**Fig. 5:**
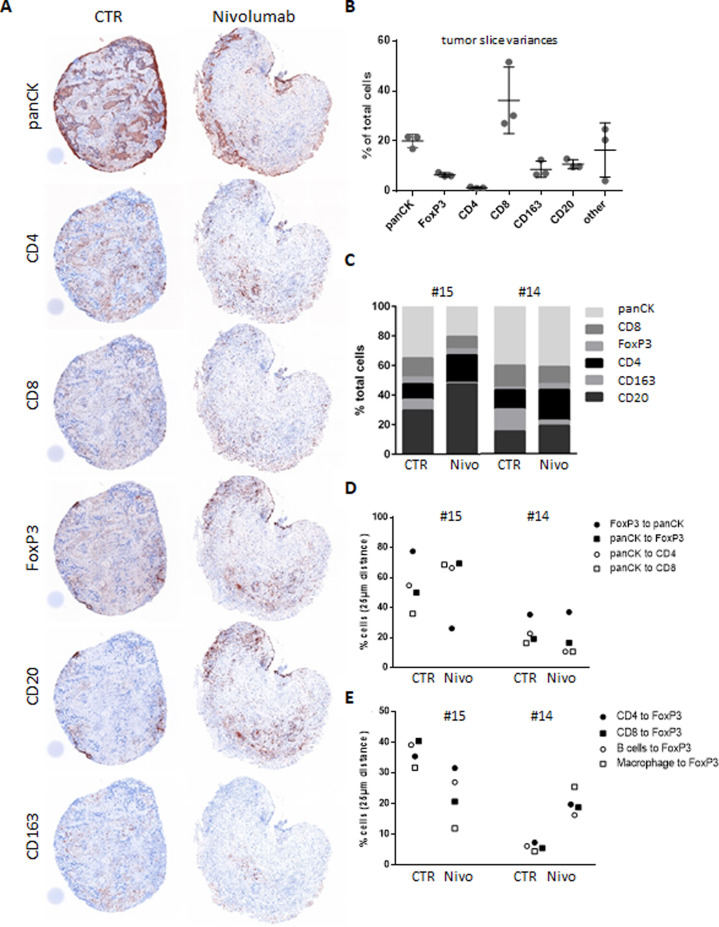
Multispectral imaging (MSI) analysis discriminates responding tissues. Tissue was stained with Ab against CD3, CD8, CD163, CD20, Foxp3, and panCK and was evaluated by MSI. **A** Representative staining of the single antibody upon conversion of the multispectral image into a pathological DAB-like view. **B** Three tissue pieces from the same patient were stained and evaluated for the percentages of the different cell populations. **C** The frequency of the different immune populations as well as tumor cells within the tissue are shown for the responder and nonresponder samples treated or not with nivolumab for 5 days. **D, E** The spatial organization of tumor cells and immune cells was evaluated by calculating the frequency of cells having a cell from another phenotype within a 25 μm radius. Data are shown for relationship involving panCK+ cells (**D**) or FoxP3+ cells (**E**).

To further investigate the immunological response in adenocarcinoma tissues, multispectral imaging was conducted (Fig. 5). Evaluation of three baseline slices from the same tumor underlined a good reproducibility of the technique as shown by almost identical frequencies for the majority of the evaluated populations (Fig. 5B). The NSCLaC specimens #14 (nonresponder) and #15 (responder) were quantitatively analyzed for the effects of nivolumab treatment (Fig. 5C). The frequency of panCK+ tumor cells was unaltered in the NR specimen, whereas it was reduced in response to nivolumab in the responder #15. Regarding the immune cells an expansion of B cells and CD4+ T cells in response to nivolumab was found in both tissues, even if it was stronger in the responder. CD8+ T cells as well as CD163+ macrophages were slightly reduced in both cases, whereas the Treg population showed an augmentation despite low absolute numbers of FoxP3+ cells.

Evaluation of the reciprocal distribution of tumor and immune cells upon nivolumab treatment highlighted major changes. Whereas the nonresponding tissue (#14) did not display changes in the relative distribution of T cells with respect to tumor cells, the tumor cells have more Treg, CD4, or CD8 T cells in their proximity (Fig. 5D) in the responding tissue #15. In contrast despite constant numbers in the tissue, Treg were less frequently in the proximity of a tumor cell (Fig. 5D). In addition, in the responding patient less Treg have other immune cells in their proximity, whereas Treg were more close to them in the nonresponding tissue (Fig. 5E). Thus, the spatial distribution of immune cells was influenced by nivolumab treatment demonstrating a reorganization of the TME.

### Clinical correlation

**Fig. 6:**
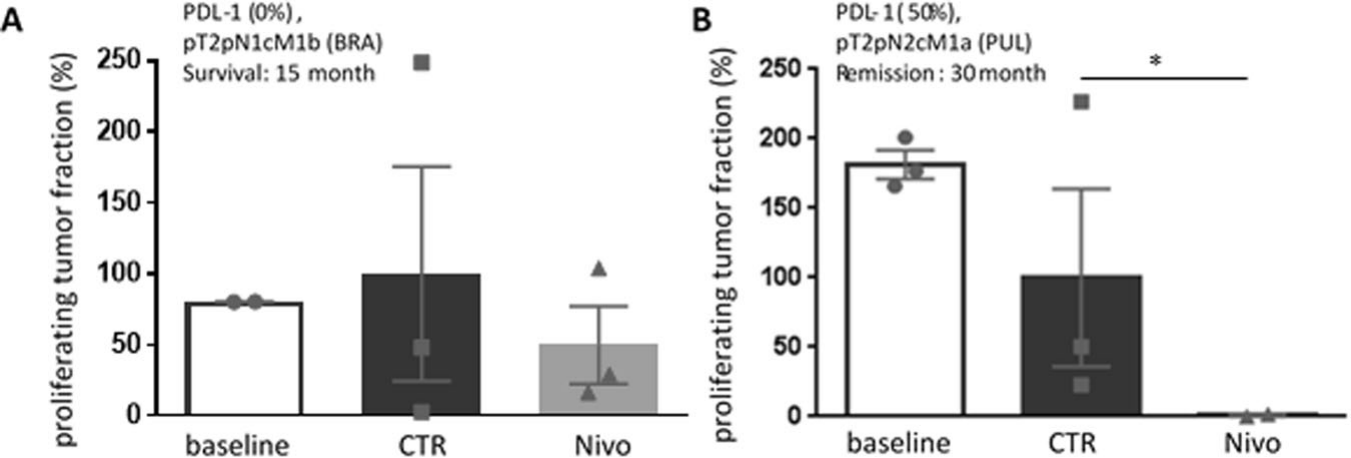
NSCLaC tissue culture susceptibility to nivolumab correlates with patients’ clinical response. Tumor specimens from two patients, **A** a clinical nonresponder and **B** a clinical responder, were treated ex vivo with nivolumab (Nivo). Cell proliferation was evaluated after 5 days. *p < 0.005 Mann–Whitney test.

For two of the specimen the clinical outcome of patients after 30 months receiving nivolumab treatment was available and thus could be compared to the ex vivo results (Fig. 6). The tissue from one patient (Fig. 6A) did not respond to nivolumab treatment as evaluated by unchanged proliferation of the tumor fraction, this patient died 15 months after surgery. In contrast, the tissue from another patient, shown in Fig. 6B, demonstrated a histological reaction to nivolumab ex vivo with a loss of proliferating tumor cells. This patient is still alive 30 months after surgery and nivolumab treatment.

## Discussion

The biological variety of heterogeneous tumor populations and the stromal cell composition as well as the diverse TME are significant attributes of each individual cancer and with the rise of possible applicable therapeutic approaches, stratification and prognostic markers are needed to improve patients’ outcome. Here, dose-dependency, individual response and clinical correlation of an ex vivo tissue culture model, using chemotherapy and an immune-checkpoint inhibitor against PD-1 show the potential for clinical patient stratification.

The implemented culture of NSCLC tissue maintained the cellular composition and architecture up to 12 days ex vivo. So far, tissue cultures and explant cultures of various entities as well as PDX models have been described in literature, reporting a well maintained viability of tumor cells [9, 12, 18, 20–22]. Adjacent tissue culture of NSCLC however was found to undergo major reorganization as the stromal and the epithelial compartment strongly proliferated. By applying cytotoxic and immune modulating drugs the effect of chemotherapy in adjacent tissue is not comparable to conditions of an intact organ, but the adjacent cultures clearly demonstrated the target of the respective drug [23, 24].

Immune related adverse effects provoked by immunotherapy is one of the major side effects and is not fully understood yet [25]. Antibody IgG4 controls were immunohistochemically investigated and revealed individual responses to IgG4 application. Although human IgG4 isotype antibodies have overall reduced effector functions, the status of fucosylation in cancer, however, might interfere and rise adverse effects [26]. Since the tissue is very limited, the relevance of IgG controls needs to be considered dependent of the research question.

Here, we focused on the immunological reaction of tumor infiltrating lymphocytes, as the TME regulate immune-checkpoint proteins, such as PD-1, CTLA-4, Tim-3, and Lag-3 [27]. Application of the clinical relevant PD-1 inhibitor nivolumab reduced the tumor vitality in 30% of the ex vivo specimens while about 20% is clinically expected [7]. TME markers responsible for a pro- or antitumor environment are for example hypoxia and soluble factors as well as the cellular and extracellular components of the tumor stroma [28–30]. Neoantigens or somatic mutational events in malignant cells may trigger inflammatory responses leading to local PD-L1 expression and are related with clinical susceptibility and immune-checkpoint stratification [7, 31]. Still, clinical data show that some PD-L1-negative patients also respond to PD-1 inhibitors [7]. In two tissue specimens low expression of PD-L1 was related to tissue susceptibility; however, high expression did not automatically correspond to tissue response. Further, the result of two tissue specimens could be followed up by clinical correlation after 30 months of first PD-1 inhibition and demonstrated the predictive value of the tissue model.

Beside PD-L1 expression high levels of TILs are regarded as a prognostic biomarker in NSCLC [29, 32]. The lymphocyte population at the tumor site might be limited as the local expansion is under discussion [25, 32]. The number of available T-lymphocytes was accordingly increased by adding autologous lymphatic tissue to the cultured NSCLC. However, the effect of PD-1 blockage did not influence individual tissue susceptibility ex vivo. Tissue lymphocytes are a highly clonal population that do not re-enter the circulation, suggesting that the pre-existing TME shape lymphocyte effectiveness [19, 27, 33].

To investigate the response of Tregs and cytotoxic T cells we analysed the cultured specimens by flow cytometry. In whole blood samples of tumor patients, PD-1 inhibition did induce a shift towards a pro-inflammatory Th1/Th17 and showed a suppression of Th2 response in prostate cancer and melanoma patients [34]. Reduced populations of Th17 cells were observed in the tissues responding to PD-1 inhibition. The compensatory high Th1 population might, however, provide a reasonable explanation, as it was shown that Th17 cells can transit from an IL-17 producing cell to an IFN-γ-producing cell called ex-Th17 cells [35]. Another study found that IFN-γ-mediated neuropilin1 (Nrp1) expression of Tregs and Nrp1 deficiency correlates with susceptibility for PD-1 inhibition [36]. Yet, Treg analysis showed contrary alterations in the responding tissues of NSCLaC and sNSCLC although Tregs were found as prognostic marker for lung cancer [37]. Differentiating between the size and granularity of lymphocytes as abnormal lymphocyte shapes are often found in close proximity to tumor cells, a higher Treg proportion could be observed. Further evidence exists that Treg characteristics seem to determine tissue response, not evaluated in the current study [36, 38].

The NSCLaC specimens were additionally investigated by multispectral imaging (MSI), recapitulating tissue response. MSI was also indicated as a suitable method to investigate explant cultures and is highly effective to investigate tissue specimens [15, 31, 39, 40]. A major B-cell expansion after PD-1 inhibition was observed in the analysed responding tissue. B cells express PD-1 and have direct pro and antitumor activities [41–44]. However, confirming our data, nonprogressing patients showed a humoral immune response after immune-checkpoint inhibition [45, 46]. Further it could be shown that the spatial relation between immune cells and tumor cells or Treg cells could discriminate the responding tissue. It is suggested that the spatial relation and to a lesser content the quantitative amount of immune cells is relevant for susceptibility determination. In line with previous studies, we found that CD8+ T cells were more in close relation to tumor cells and less close to Tregs specifically in the tissue responding to PD-1 inhibition ex vivo [47, 48].

Taken together, standardized patient-derived tissue culture was shown for the first time to enable detailed analysis of individual adaptation to multiple biologicals. This model displays an orchestrated interaction of all present mutations and influencing cellular components. Especially biological relevant T-cell adaptations were found, confirming studies that investigated clinical specimens of larger cohorts. This unique ex vivo approach allows not only to determine clinical relevant responses but also to investigate relevant questions regarding tumor susceptibility and regulation.

## Materials and methods

### Specimens

Tumor specimens were obtained from patients treated at the university hospital in Leipzig, Germany. A total of 15 NSCLC patients were included in this study (supplementary Table 1), which was approved by the Ethics Committee of the Medical Faculty, University of Leipzig (AZ 370-1316122013, AZ 370-13-ff). All patients provided their informed written consent to this study.

### Preparation of tissue slice cultures

The preparation protocol was previously [12] described and applied with some modifications. In brief, immediately after surgical resection and first macroscopic pathological assessment, tumor samples were cut into slices of 350 μm using a tissue chopper (McIlwain TC752; Campden Instruments, Lafayette, USA). Tissue slice diameter was standardized by using a 3 mm coring tool (kai Europe, Solingen, Germany). Three tissue slices were randomly pooled, placed on membrane inserts (Millipore Corporation, Billerica, USA) and cultivated in six-well plates. Slices were incubated under standardized conditions of 37 °C and 5% CO_2_. Medium (phenol-free RPMI 1640 (Thermo Fisher scientific, Waltham, USA), supplemented with 1% penicillin/streptomycin (Merck, Darmstadt, Germany; 10 000 U penicillin /10 mg/ml streptomycin in 0.9% NaCl), 1% L-glutamine (Thermo Fisher Scientific, 200 mM), 10% fetal calf serum (Thermo Fisher Scientific) was changed every other day after preparation unless stated otherwise. Slices were fixed overnight using 4% paraformaldehyde (PFA) at the day of preparation (baseline) and after cultivation. Untreated cultivated slices served as controls (CTR). In experiments using nivolumab, IgG4 controls were used when possible.

### Experimental setup and treatment

Twenty four hours after tumor resection, the cultured slices were treated with different drugs. First, slice cultures of six specimens were treated with cisplatin (1, 10 µM) or paclitaxel (1, 10, and 100 nM) (medac GmbH, Germany) for 72 h to investigate dose-response and individual susceptibility. Ten micromolar of cisplatin and 100 nM of paclitaxel were selected for further studies. Tumor specimen from 12 patients were treated with 3 µg/ml of Nivolumab (opdivo; Bristol Myers-Squibb, USA), the same concentration currently used in murine as well as clinical settings. Drug containing medium was prepared at the day of treatment and changed every other day. Control conditions were cultivated as indicated above without cytotoxic supplement and when possible with IgG4 (cat.No. bgal-mab114, invivogen, France) as an isotype control to Nivolumab.

### Flow cytometry

A combination of mechanical and enzymatic dissociation protocol was applied for the generation of singe cells from the tumor slices. Briefly, cultures were minced with a surgical scalpel into small pieces of ~1–2 mm. The tissue pieces were then incubated for 2 × 20 min with Liberase (Roche, Swiss) at 37 °C and then poured over a 70 µm mesh. Cells were washed once with PBS 5 min at 400 × *g*, followed by incubation for 30 min with fixable viability Dye eFluor 780 (LD), and an additional 20 min incubation with the Fc-block. After a washing step fluorescence labelled antibodies (anti-human CD3, UCHT1, PE-Cy7; anti-human CD4, RPA-T4, APC; anti-human CD8, HIT8a, FITC; combined with anti-human CD127, HIL-7R-M21, PE and anti-human CD25, MA251, BV421, or with anti-human CD183/CXCR3, 1C6, PE and anti-human CD196/CCR6, 11A9, BV421 all from BD) were applied for 20 min to identify T-cell populations. The gating strategy is shown in supplementary Fig. 1. Cells were kept on ice and in dark during the staining process. Cells were measured and analysed using an LSR II flow cytometer. FACSDiVa 6.1.3 software was used for the measurement and analysis. FlowJo v10.2 was used for documentation and analysis.

### Staining procedure and analysis

PFA (4%) fixed slices were embedded in paraffin and processed to 5 μm sections. Hematoxylin and eosin staining (HE) was performed to assess histopathological aspects and tumor cell proportion. Overall cell count, tumor cell count, and proliferation were analyzed by immunofluorescent staining. In brief, after antigen retrieval sections were washed with 0.3% PBS/TritonX and blocked with 5% normal goat serum (Sigma-Aldrich, USA) for 30 min. Primary antibodies against cytokeratins (AE1 + 3, mouse; BioGenex, USA), Ki67 (rabbit, DCS, Germany) and cleaved PARP (rabbit, Abcam, UK), CD3 (rat, Serotec, USA), CD8 (mouse, Cell Signaling, USA), PD-L1 (rabbit, Abcam), or FoxP3 (mouse, Invitrogen, USA) were diluted in 0.5% BSA and incubated at 4 °C overnight. Sections were rinsed with 0.3% PBS/TritonX and labeled with the secondary antibodies goat-anti-rabbit 568 or 647, goat-anti-mouse 568 or 647, goat-anti-rat 647 (Alexa Fluor, Invitrogen, USA), respectively. Nuclei were stained with Hoechst 33342 (Aldrich). Tumor-cell-containing areas were controlled in consecutive HE sections by a pathologist using slide scans (Pannoramic SCAN and Pannoramic Viewer, 3D Histech, Budapest, Hungary) to investigate varying tumor cell fractions (TF).

Multispectral imaging (MSI) was performed as previously described [49] using following antibodies: CD20 (L26, Dako), CD8 (SP16, Abcam), Foxp3 (236 A/E7, Abcam), CD3 (SP7, Thermo Fischer), CD163 (MRQ-26, Cell Marque), and pan cytokeratin (panCK, AE1/AE3, Dako). The Opal fluorophores were acquired from Akoya biosciences (Marlborough, USA). For analyses whole-tissue sections were acquired and analyzed with the inform software (Akoya bioscience). The different cell populations were determined as follow: tumor cells as panCK^+^, macrophages as CD163^+^, CD8 T cells as CD3^+^ CD8^+^, Treg as CD3^+^ Foxp3^+^, CD4 T cells as CD3^+^ Foxp3^neg^ CD8^neg^, and B cells as CD20^+^. Cells with no specific staining are in the “other” category.

### Statistical analysis

The total cell count (Hoechst positive), tumor cell count (Hoechst and cytokeratin positive), and proliferating tumor cell count (Hoechst, cytokeratin and Ki67 positive) was acquired for every picture. Mean slice values were then calculated to obtain the mean value for each condition and are displayed in the figures. Either parametric or nonparametric statistical tests were performed using GraphPad Prism 6 (GraphPad Software, La Jolla, USA), depending of data size and distribution. *P* < 0.05 was considered significant.

## Supplementary information

Gating strategy

Suppl. figure legend

## Data Availability

All data generated or analysed during this study are included in this published article and its supplementary information files.
