# Supplementary figures and images for: Human tissue cultures of lung cancer predict patient susceptibility to immune-checkpoint inhibition
Source: Cell Death Discov. 2021 Sep 25;7:264. doi: 10.1038/s41420-021-00651-5 (PMC8464600; doi:10.1038/s41420-021-00651-5)

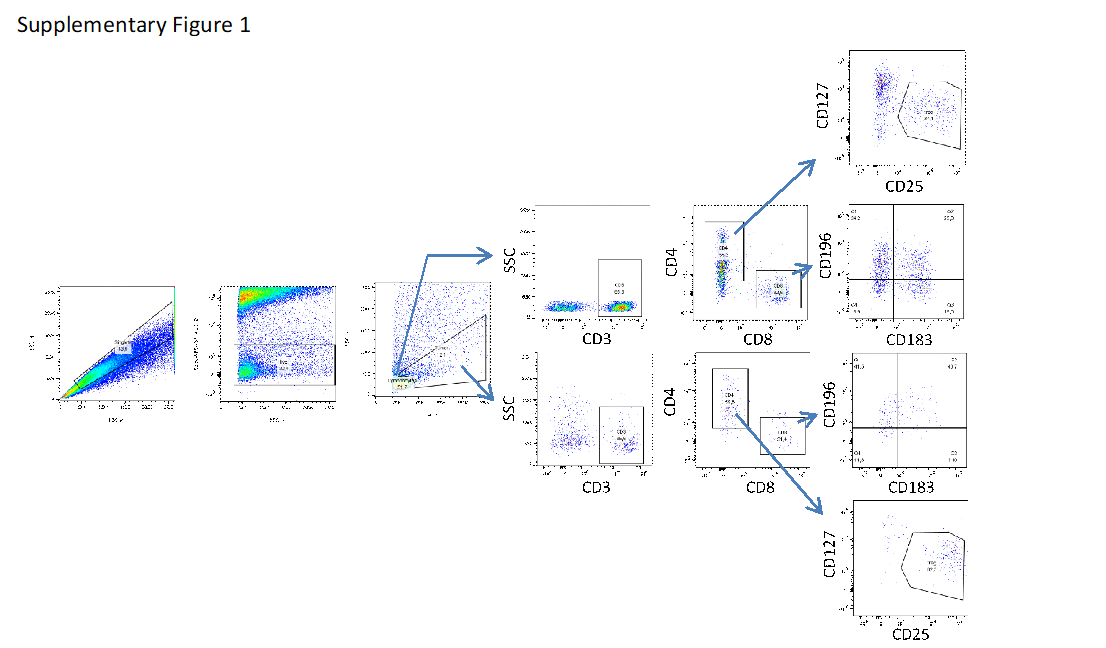

Supplement: Supplementary file 1 — Gating strategy [file 41420_2021_651_MOESM1_ESM.tif]
