# Supplementary material for: Human tissue cultures of lung cancer predict patient susceptibility to immune-checkpoint inhibition
Source: Cell Death Discov. 2021 Sep 25;7:264. doi: 10.1038/s41420-021-00651-5 (PMC8464600; doi:10.1038/s41420-021-00651-5)
Supplement: Supplementary file 2 — Suppl. figure legend [file 41420_2021_651_MOESM2_ESM.docx]

**Supplementary Figure 1: Gating strategy for T-cell identification and sub-typing.** Shown is the gating strategy used for the evaluation of the tumor infiltrate by flow cytometry. First of all, single cells were identified using the Area versus Height value of the FSC parameter and live cells for their negative staining to the Fixable Viability Stain. Lymphocytes were subdivided into big and small populations based on scatter parameters SSC-A and FSC-A. CD3^+^ T cells were subdivided into CD4^+^ and CD8^+^ T cells. Within the CD4^+^ T cells staining for CD196 and CD183 was used to discriminated Th1 (CD183^+^), Th17 (CD196^+^) and Th2 (CD196^neg^ CD183^neg^) cells, whereas Treg were identified as CD25^+^ CD127 ^low/neg^ cells
